# Association between Sleep Duration and Left Ventricular Hypertrophy for Patients with Type 2 Diabetes Mellitus

**DOI:** 10.1155/2023/5532778

**Published:** 2023-11-28

**Authors:** Lin Mu, Chao Li, Wenying Zhao, Aihua Li, Dong Zhao, Baoyu Zhang

**Affiliations:** ^1^Center for Endocrine Metabolism and Immune Diseases, Beijing Luhe Hospital, Capital Medical University, Beijing 101149, China; ^2^Beijing Key Laboratory of Diabetes Research and Care, Beijing 101149, China; ^3^Department of Cardiology, Beijing Luhe Hospital, Capital Medical University, Beijing 101149, China

## Abstract

**Objective:**

In this study, we aimed to estimate the impact of sleep duration on left ventricular hypertrophy (LVH) in type 2 diabetes mellitus (T2DM).

**Methods:**

Consecutive patients with T2DM undergoing transthoracic echocardiography (TTE) in our center from October 2017 to February 2021 were analyzed. The association of the risk of LVH in T2DM patients was evaluated using univariable and multivariable logistic regression analyses.

**Results:**

This study finally included 2689 adult patients (mean age 51.8 ± 12.5 years, 56.2% men, mean sleep duration 7.6 ± 1.4 hours per day). Of all patients, 655 (24.4%) patients were diagnosed with LVH and 2034 did not have LVH. All patients were adults and were diagnosed with T2DM. In the univariate and multivariate regression analyses, gender, sleep duration, body mass index (BMI), waist, hemoglobin (Hb), blood creatinine (Cr), and high-density lipoprotein cholesterol (HDL-c) were associated with LVH. In the restricted cubic spline (RCS) model, the cut-off points of sleep duration given refer to the group of patients with T2DM and LVH were 8 hours per day. With the cut-off points, the multivariable analysis demonstrated that, for diabetic patients, LVH was significantly correlated with a sleep duration of 8 hours per day, hemoglobin, blood urea nitrogen (BUN), and HDL-c.

**Conclusion:**

For patients with T2DM, long sleep duration (>8 hours per day), hemoglobin, BUN, and HDL-c were independently associated with LVH. This trial is registered with NCT03811470.

## 1. Background

Previous studies have shown an increasing incidence and prevalence of diabetes [1–3]. As reported in Denmark, the overall prevalence of diabetes in 2016 was 4.4% for type 2 diabetes mellitus (T2DM), with annual increases for T2DM since 1996 of 5.5% [1]. In Iceland, the prevalence of T2DM more than doubled in the period of 2005–2018 [2]. In China, in 2018, the prevalence of diabetes was about 12.4% and the estimated prevalence of prediabetes was 38.1% [3].

Diabetes has caused a tremendous economic burden in world. The estimated global direct health expenditure on diabetes in 2019 is USD 760 billion and is expected to grow to a projected USD 825 billion by 2030 and USD 845 billion by 2045 [4]. In the United States, the total cost of diabetes in 2017 was USD 327 billion and more than half of the expenditure was attributable to T2DM [5]. Patients in the United States with diabetes incurred average medical expenditures of about USD 16,750 per year [4]. China had one of the highest estimated total costs of diabetes with USD 109.0 billion [4].

Diabetes is a progressive disorder causing numerous complications, including small vessel or microvascular disease and large vessel or macrovascular disease [6]. Microvascular complications could affect the kidney, retina, and peripheral nerves termed as diabetic nephropathy, diabetic retinopathy, and diabetic neuropathy, respectively [7]. The macrovascular complications that affect the heart are identified as cardiovascular disease, the complications affecting the peripheral arteries are termed as peripheral vascular disease, and those affecting the brain are termed as cerebrovascular disease [8]. Cardiac hypertrophy is one of the manifestations of diabetic cardiomyopathy [9]. Metabolic disturbances caused by diabetes could promote cardiac remodeling, fibrotic diastolic dysfunction, and, ultimately, decreased ejection fraction [10].

Sleep is a public health concern affecting up to one-third of the population, and a large body of evidence has proved the association between insufficient sleep duration and quality and the risk of obesity, insulin resistance, and T2DM [11]. As reported, sleep disorders are highly prevalent among patients with T2DM [12]. Previous studies have proved a U-shaped association between sleep duration and incidence of T2DM, reporting 7-8 hours of sleep per night as the lowest risk [13]. In observational studies, short sleep duration was associated with both a higher risk of incidence of T2DM and may also predict worse outcomes in those with existing diabetes [12]. Experimental and clinical studies have suggested an independent association between obstructive sleep apnea (OSA) and left ventricular mass (LVM), but there are few studies evaluating the impact of sleep duration on the LVM for diabetic patients.

In this study, we aimed to estimate the impact of sleep duration on the LVM for T2DM patients.

## 2. Methods

### 2.1. Participants

This study is a large-scale single-center retrospective study performed at the Beijing Luhe Hospital. From October 2017 to February 2021, we retrospectively reviewed 3074 consecutive patients undergoing transthoracic echocardiography (TTE) in the Center for Endocrine Metabolism and Immune Disease. This study was registered at clinicaltrials.gov with the registry name “Serological, Metabolomics and Genomics Studies of Metabolic Diseases” and allotted the identifier NCT03811470. This study complied with the tenets of the Declaration of Helsinki. All patients gave their informed consent prior to their inclusion in the study.

The inclusion criteria for this study were as follows: all participants were diagnosed with T2DM. All participants were adults. Participants underwent a cardiac ultrasound, and detailed cardiac measurements were recorded. Participants had detailed health records.

The exclusion criteria for this study were as follows: patients <18 years of age were excluded. Patients with structural heart disease, valvular heart disease, hypertrophic cardiomyopathy, or undergoing cardiac surgery were excluded.

### 2.2. Data Collection

All clinical data were acquired via a search of the Hospital Information System. The information retrieved included age, gender, blood pressure (BP), heart rate (HR), height, weight, body mass index (BMI), diabetes duration, smoke history, alcohol intake history, hemoglobin (Hb), fasting blood glucose, hemoglobin A1C (HbA1c), blood lipid level, aminotransferase level, thyroid function and history of hypertension, hyperlipidemia, hyperuricemia (HUA), gout, coronary heart disease (CHD), and vascular disease. As reported, sleep duration was assessed by asking participants “What time do you usually go to bed? When get up?” and we calculated the sleep duration based on the results [14]. Left ventricular hypertrophy (LVH), which can be measured by TTE, was represented by the left ventricular mass index (LVMI).

LVM was calculated according to the following formula [15]:

(1) $$\begin{array}{c} \text{LVM}=0.8*1.04*{\left(\text{IVSD}+\text{LVEDD}+\text{LVPWD}\right)}^{3}-{\text{LVEDD}}^{3}+0.6,\\ \text{LVMI}=\frac{\text{LVM}}{\text{BSA}}, \end{array}$$

IVSD: interventricular septum diameter; LVEDD: left ventricular end-systolic diameter; LVPWD: left ventricular posterior wall diameter; BSA: body surface area.

As recommended, LVH was defined as a LVMI of more than 102 g/m^2^ for males or 88 g/m^2^ for females [16].

### 2.3. Statistical Analysis

**Figure 1:**
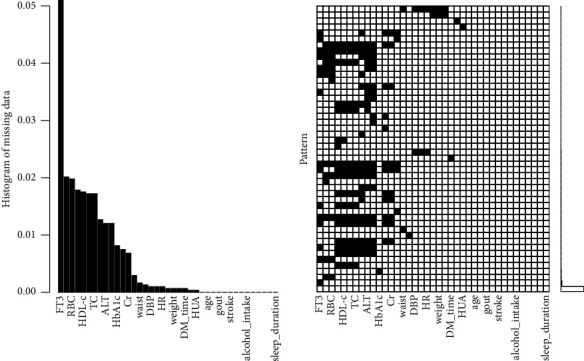
Distribution of missing data in the study. In this study, the proportion of missing variables was not more than 5%. The variable with the most missing is FT3 (∼5.0%). FT3, serum-free triiodothyronine; RBC, red blood cell; HDL-c, high-density lipoprotein cholesterol; TC, total cholesterol; ALT, alanine aminotransferase; HBA1c, hemoglobin A1C; Cr, creatinine; DBP, diastolic blood pressure; HR, heart rate; DM, diabetes mellitus; HUA, hyperuricemia.

As shown in Figure 1, the proportion of missing variables were not more than 5%, so participants with missing data were included. Finally, 2689 T2DM patients were included in the study.

Continuous variables were presented as the mean ± standard deviation (SD) or medians (interquartile ranges) and compared by Student's *t*-tests or Mann–Whitney *U* tests. Categorical variables were presented as number (proportion) and compared by Pearson's *χ*^2^ test. Comparisons were made between the variables in patients with LVH and the control group. A *P* value of less than 0.05 was considered significant.

The association of the risk of LVH in patients with T2DM was evaluated using univariable logistic regression analysis. The variables with a *P* value of less than 0.1 were entered into the backward regression multivariable analysis.

## 3. Results

A total of 2689 T2DM patients were finally included in the study. Out of the 2689 patients, 655 (24.4%) patients were diagnosed with LVH and 2034 patients did not have LVH, who formed the control group. Baseline demographic information is provided in Table 1. The mean age was 51.8 ± 12.5 years; 56.2% were men; the mean BMI was 26.7 ± 4.0 kg/m^2^; the mean waist measurement was 94.3 ± 10.8 cm; the mean HR was 83.9 ± 12.6 bpm; the mean systole blood pressure (SBP) was 132.6 ± 17.7 mmHg; the mean diastolic blood pressure (DBP) was 80.0 ± 11.9 mmHg; the mean sleep duration was 7.6 ± 1.4 hours per day. Of the participants, 1131 (42.1%) patients had hypertension, 1214 (45.1%) patients had hyperlipidemia, 312 (11.6%) had HUA, 59 (2.2%) had gout, and 347 (12.9%) had CHD. Other clinical characteristics of patients are summarized in Table 1.

Patients with LVH were older than the control group (54.1 ± 12.1 vs. 51.2 ± 12.5 years, *P* < 0.001) and had a longer duration of diabetes (88 (18–165) vs. 62 (7–133) months, *P* < 0.001). Patients with LVH slept more than the control group (7.8 ± 1.3 vs. 7.5 ± 1.4, *P* < 0.001). Compared with the control group, patients who were diagnosed with LVH had a lower weight, lower height, lower BMI, lower waist, lower BP (SBP and DBP), lower triglyceride (TG), lower high-density lipoprotein cholesterol (HDL-c), lower low-density lipoprotein cholesterol (LDL-c), lower left ventricular ejective fraction (LVEF), higher Hb, higher alanine transaminase (ALT), higher creatinine (Cr), higher blood urea nitrogen (BUN), and higher uric acid (UA). Patients with LVH showed a higher prevalence of HUA and gout (*P* < 0.05), while there were no significant differences in the history of hypertension, hyperlipidemia, or CHD.

All variables entered multivariable analysis with a *P* value less than 0.1. As shown in Table 2, after univariable analysis, the variables of age, BMI, SBP, DBP, waist, diabetes duration, Hb, ALT, BUN, Cr, UA, HDL-c, serum-free triiodothyronine (FT3), gender, HUA, gout, smoking, smoking history, alcohol drinking, history of drinking, and sleep duration were entered into the multivariable logistic regression analysis. The multivariable analysis demonstrated that LVH was significantly correlated with BMI (OR = 0.93, *P* = 0.001), waist (OR = 0.97, *P* < 0.001), Hb (OR = 1.008, *P* = 0.012), Cr (OR = 1.015, *P* < 0.001), HDL-c (OR = 0.5, *P* < 0.001), gender (OR = 0.215, *P* < 0.001), and sleep duration (OR = 1.093, *P* = 0.022) for T2DM patients (Table 2).

**Figure 2:**
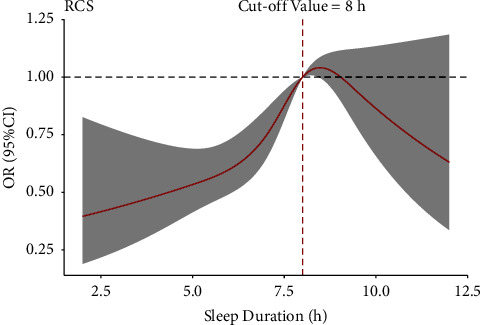
The cut-off points of sleep duration for LVH.

As shown in Figure 2, a restricted cubic spline (RCS) model was used to obtain the cut-off point of sleep duration for LVH. The cut-off point of sleep duration was 8 hours.

With the cut-off point, sleep duration was converted into rank variables. The followed multivariable analysis demonstrated that LVH was significantly correlated with a sleep duration of 8 hours per day (OR = 1.563, *P* < 0.001), Hb (OR = 1.009, *P* = 0.001), BUN (OR = 1.093, *P* = 0.002), and HDL-c (OR = 0.612, *P* = 0.001) for T2DM patients (Table 3).

## 4. Discussion

In the present study, we conducted a single-center, observational, retrospective study. A total of 2689 consecutive patients with T2DM, for whom transthoracic echocardiography (TTE) was performed and LVMI was calculated, were included in the study. Compared with the control group, patients with LVH were older and had a longer sleep duration per day. In the RCS model, the cut-off point of sleep duration was 8 hours per day. Multivariable analysis demonstrated that LVH was significantly correlated with a sleep duration of 8 hours per day (OR = 1.563, *P* < 0.001), Hb (OR = 1.009, *P* = 0.001), BUN (OR = 1.093, *P* = 0.002), and HDL-c (OR = 0.612, *P* = 0.001) for T2DM patients.

To our knowledge, this is the first study evaluating sleep duration for the prediction of LVH. As a complication of common cardiovascular disorders (such as coronary atherosclerosis and myocardial infarction) and metabolism syndrome, LVH, which could progress to heart failure, needs early prevention and treatment [17–19]. Although many drug therapies, nutraceutical and dietary measures are available for the management of ventricular hypertrophy, most patients nonetheless experience a downhill course [20]. Positive changes in lifestyle, a safe and inexpensive measure, may have merit in the management of ventricular hypertrophy, while the impact of sleep duration on ventricular hypertrophy remains to be fully understood. In the present study, we found that sleep duration may not be as long as possible, and a sleep duration longer than 8 hours may be one of the risk factors for ventricular hypertrophy.

Our study has several strengths. First, we conducted the study among a relatively high number of individuals (*n* = 2689). Second, we used RCS analysis to search the optimal cut-off point rather than the cut-point recommended in the guidelines, as the cut-off point of sleep duration to LVH may be unique. Third, the model was adjusted for duration of diabetes, as diabetes' duration may be associated with LVH.

Previous studies had proved that a long sleep duration was associated with an increased risk of all-cause mortality and cardiovascular events [21, 22].

Several potential mechanisms may contribute to the association between long sleep duration and LVH. First, getting too much sleep can be a risk factor for hypertension, and hypertension is an important risk factor for cardiac hypertrophy. Sleep duration and BP are linked in a complex way. After adjusting for confounders such as age, BMI, and alcohol consumption, a *U*-shaped curve was reported between sleep duration and the risk of hypertension [23]. Sleep duration more than or equal to 9 hours was a risk factor of hypertension, and the same result was found for sleep duration more than or equal to 10 hours [24]. Second, long sleep duration may be a representation of OSA, a known cause of increased need for sleep and an identified risk factor for ventricular hypertrophy [25]. Patients with OSA may be at risk for LVH because of their poorer quality of sleep, such that they sleep for longer periods of time, more often than not in a refreshing sleep state, and often with an early onset of headache [26]. Third, spending a long time in bed may be linked to sleep fragmentation [27], which was associated with more severe arteriolosclerosis and LVH. Changes in inflammatory markers (such as C-reactive protein (CRP), interleukin-6 (IL-6), and tumor necrosis factor-*α* (TNF-*α*)) are associated with long sleep duration, which may contribute to ventricular hypertrophy [28]. In the present study, we found that HDL-c was negatively associated with LVH. Schillaci et al. found that low HDL-c was an independent predictor of LVM in untreated hypertensive patients [29], which is consistent with our results. Previous studies have proved that premenopausal women, as compared to men, are less prone to develop ventricular hypertrophy, while this protection is lost after menopause [30, 31]. In our study, being female was an independent predictor of LVH for T2DM patients, as the participants in our study were older, with a mean age of 51.8 ± 12.5 years, and most female patients were postmenopausal.

Several limitations of our study should be acknowledged. First, all periods of sleep duration were self‐reported by questionnaire or interview instead of detection using sleep monitoring equipment. Second, factors such as sleep apnea are an independent predictor of the risk of LVH but were not assessed in our study. Third, this is a single-center study, and any extrapolation of the conclusions may be limited.

## 5. Conclusion

For patients with T2DM, a long sleep duration (>8 hours per day), Hb, BUN, and HDL-c were independently associated with LVH.

## Figures and Tables

**Table 1:** Baseline characteristics of patients.

Variables	All *N* = 2689	Control *N* = 2034	LVH patients *N* = 655	*P* value
Age (years)	51.8 ± 12.5	51.2 ± 12.5	54.1 ± 12.1	<0.001
Male	1510 (56.2%)	1330 (65.4%)	180 (27.5%)	<0.001
Height (cm)	165.9 ± 8.7	167.5 ± 8.5	160.8 ± 7.6	<0.001
Weight (kg)	73.8 ± 14.3	76.6 ± 13.9	65.2 ± 11.6	<0.001
BMI (kg/m^2^)	26.7 ± 4.0	27.2 ± 4.0	25.1 ± 3.7	<0.001
Waist (cm)	94.3 ± 10.8	95.9 ± 10.4	89.4 ± 10.6	<0.001
HR (bpm)	83.9 ± 12.6	83.9 ± 12.8	83.5 ± 12.1	0.440
SBP (mmHg)	132.6 ± 17.7	133.3 ± 17.6	130.5 ± 18.1	<0.001
DBP (mmHg)	80.0 ± 11.9	80.8 ± 11.8	77.0 ± 11.7	<0.001
Sleep duration (hours/day)	7.6 ± 1.4	7.5 ± 1.4	7.8 ± 1.3	<0.001
Diabetes duration (months)	66 (10–142)	62 (7–133)	88 (18–165)	<0.001
History of hypertension	1131 (42.1%)	855 (42.0%)	276 (42.1%)	0.963
History of hyperlipidemia	1214 (45.1%)	923 (45.4%)	291 (44.4%)	0.671
History of HUA	312 (11.6%)	258 (12.7%)	54 (8.2%)	0.002
History of gout	59 (2.2%)	53 (2.6%)	6 (0.9%)	0.010
History of CHD	347 (12.9%)	259 (12.7%)	88 (13.4%)	0.641
History of stroke	134 (5.0%)	97 (4.8%)	37 (5.6%)	0.368
History of vascular disease	128 (4.5%)	97 (4.8%)	31 (4.7%)	0.970
Smoking	841 (31.3%)	721 (35.4%)	120 (18.3%)	<0.001
Smoking history	391 (14.5%)	326 (16.0%)	65 (9.9%)	<0.001
Alcohol drinking	1036 (38.5%)	891 (43.8%)	145 (22.1%)	<0.001
History of drinking	874 (32.5%)	740 (36.4%)	134 (20.5%)	<0.001
HbA1c (%)	8.6 (7.2–10.4)	8.6 (7.2–10.4)	8.7 (7.1–10.6)	0.756
Hb (g/L)	146 (135–157)	145 (134–157)	149 (138–159)	<0.001
Blood glucose (mmol/L)	8.2 (6.6–10.9)	8.2 (6.6–10.9)	8.3 (6.7–10.8)	0.427
ALT (U/L)	21 (15–34)	21 (15–33)	23 (16–36)	0.004
AST (U/L)	18 (14–24)	18 (14–24)	18 (15–24)	0.619
Cr (*μ*mol/L)	66.5 (56.0–77.0)	65 (55–76)	69 (60–81)	<0.001
BUN (mmol/L)	5.0 (4.1–6.0)	4.9 (4.0–5.9)	5.2 (4.3–6.3)	<0.001
UA (*μ*mol/L)	316 (263–380)	312 (258–375)	331 (280–394)	<0.001
TC (mmol/L)	4.76 (3.95–5.57)	4.79 (3.89–5.58)	4.64 (3.89–5.56)	0.060
TG (mmol/L)	1.55 (1.07–2.37)	1.50 (1.04–2.28)	1.68 (1.14–2.52)	<0.001
HDL-c (mmol/L)	1.15 (0.99–1.35)	1.16 (1.0–1.36)	1.12 (0.95–1.31)	<0.001
LDL-c (mmol/L)	3.05 (2.42–3.68)	3.07 (2.44–3.69)	2.97 (2.36–3.64)	0.126
TSH (*μ*IU/mL)	1.82 (1.24–2.79)	1.84 (1.25–2.83)	1.77 (1.18–2.74)	0.085
FT4 (ng/dL)	13 (11.8–14.5)	13 (11.8–14.5)	13 (11.7–14.5)	0.880
FT3 (pg/mL)	3.00 (2.69–3.33)	2.99 (2.69–3.32)	3.04 (2.71–3.35)	0.133
LVEF (%)	70 (66–73)	70 (66–74)	68 (64–72)	0.044

BMI, body mass index; HR, heart rate; SBP, systolic blood pressure; DBP, diastolic blood pressure; HUA, hyperuricemia; CHD, coronary heart disease; HBA1c, hemoglobin A1C; Hb, hemoglobin; ALT, alanine aminotransferase; AST, aspartate aminotransferase; Cr, creatinine; BUN, blood urea nitrogen; UA, uric acid; TC, total cholesterol; TG, triglycerides; HDL-c, high-density lipoprotein cholesterol; LDL-c, low-density lipoprotein cholesterol; TSH, thyroid-stimulating hormone; FT4, serum-free thyroxine; FT3, serum-free triiodothyronine; LVEF, left ventricular ejection fraction. The *P* value refers to the statistical difference between the control group and the studied group.

**Table 2:** Logistic multivariable regression analysis of factors of LVH.

Variables	*B*	S.E.	Wald	*P*	OR	95% CI
Age	0.007	0.005	1.706	0.191	1.007	0.997–1.017
DBP	−0.004	0.006	0.457	0.499	0.996	0.984–1.008
SBP	−0.003	0.004	0.676	0.411	0.997	0.99–1.004
BMI	−0.073	0.023	10.177	0.001	0.93	0.889–0.972
Waist	−0.03	0.008	12.84	<0.001	0.97	0.955–0.986
Diabetes duration	0	0.001	0.406	0.524	1.0	0.999–1.002
Hb	0.008	0.003	6.352	0.012	1.008	1.002–1.015
ALT	0.001	0.002	0.569	0.451	1.001	0.998–1.005
BUN	0.045	0.036	1.577	0.209	1.046	0.975–1.121
Cr	0.015	0.003	20.01	<0.001	1.015	1.009–1.022
UA	0.001	0.001	0.834	0.361	1.001	0.999–1.002
HDL-c	−0.693	0.189	13.422	<0.001	0.5	0.345–0.725
FT3	0.028	0.067	0.174	0.676	1.028	0.902–1.173
Gender	−1.539	0.149	107.209	<0.001	0.215	0.16–0.287
History of HUA	−0.217	0.185	1.371	0.242	0.805	0.56–1.157
History of gout	0.03	0.483	0.004	0.951	1.03	0.4–2.652
Smoking	0.139	0.152	0.84	0.359	1.149	0.854–1.547
Smoking history	0.236	0.173	1.867	0.172	1.266	0.903–1.777
Alcohol drinking	−0.05	0.156	0.102	0.75	0.951	0.701–1.292
History of drinking	−0.084	0.148	0.321	0.571	0.92	0.688–1.229
Sleep duration	0.089	0.039	5.251	0.022	1.093	1.013–1.18

DBP, diastolic blood pressure; SBP, systolic blood pressure; BMI, body mass index; Hb, hemoglobin; ALT, alanine aminotransferase; BUN, blood urea nitrogen; Cr, creatinine; UA, uric acid; HDL-c, high-density lipoprotein cholesterol; FT3, serum-free triiodothyronine; HUA, hyperuricemia.

**Table 3:** Logistic multivariable regression analysis of the risk of LVH.

Variables	*B*	S.E.	Wald	*P*	OR	95% CI
Age	−0.056	0.115	0.235	0.628	0.946	0.754–1.186
BMI	−0.031	0.018	3.031	0.082	0.969	0.936–1.004
Waist	0.005	0.007	0.490	0.484	1.005	0.991–1.018
Hb	0.009	0.003	10.673	0.001	1.009	1.003–1.014
BUN	0.089	0.029	9.277	0.002	1.093	1.032–1.157
Cr	0.004	0.003	1.951	0.163	1.004	0.998–1.009
UA	0.001	0	2.304	0.129	1.001	1.000–1.002
HDL-c	−0.490	0.154	10.155	0.001	0.612	0.453–0.828
FT3	−0.005	0.057	0.007	0.934	0.995	0.890–1.114
Gender	0.012	0.110	0.011	0.915	1.012	0.815–1.256
History of HUA	−0.081	0.147	0.307	0.579	0.922	0.692–1.229
History of gout	0.107	0.309	0.121	0.728	1.113	0.608–2.039
Smoke	0.033	0.106	0.097	0.756	1.034	0.839–1.273
History of hypertension	−0.044	0.090	0.242	0.623	0.957	0.803–1.140
History of hyperlipidemia	0.124	0.087	2.042	0.153	1.132	0.955–1.343
Alcohol drinking	0.008	0.103	0.006	0.940	1.008	0.823–1.235
Enough sleep	0.446	0.085	27.723	<0.001	1.563	1.323–1.845
Sleep quality						
Poor			2.863	0.239		
Moderate	0.523	0.317	2.726	0.099	1.687	0.907–3.138
Good	0.451	0.310	2.122	0.145	1.570	0.856–2.883

BMI, body mass index; Hb, hemoglobin; BUN, blood urea nitrogen; Cr, creatinine; UA, uric acid; HDL-c, high-density lipoprotein cholesterol; FT3, serum-free triiodothyronine; HUA, hyperuricemia.

## Data Availability

All clinical data were acquired from the Beijing Luhe Hospital. The data of this study can be obtained from the corresponding author upon reasonable request.
